# Cerebral Venous Thrombosis Presenting as a Subacute Headache in a Young Man With Undiagnosed Factor V Leiden Mutation

**DOI:** 10.7759/cureus.14665

**Published:** 2021-04-24

**Authors:** Tanvi H Patel, Ramya Bachu, Jennifer E Naylor, Gerry Ezell

**Affiliations:** 1 Internal Medicine, Baptist Health-University of Arkansas for Medical Sciences, North Little Rock, USA; 2 Graduate Medical Education, Baptist Health-University of Arkansas for Medical Sciences, North Little Rock, USA

**Keywords:** cerebral venous sinus thrombosis, hypercoagulable, factor v leiden, papilledema

## Abstract

Cerebral venous sinus thrombosis (CVT) is a rare but potentially life-threatening condition that presents with non-specific symptoms. This condition is more common in women and can be associated with local infection and hypercoagulable conditions, including protein C and S deficiency, factor V Leiden mutation, anti-thrombin III deficiency, thrombophilia, vasculitis, and malignancy. We report the case of a 24-year-old man who presented with a left temporal headache and right upper and lower extremity paresthesia. He also experienced impaired vision (altered spatial sensation), dental pain, bruxism, nausea, and vomiting. Magnetic resonance imaging and magnetic resonance venography of the brain revealed widespread thrombosis of the cerebral sinuses as well as left superior cerebral cortical veins bilaterally. No evidence of venous infarct was found. Subsequent hematologic evaluation showed the presence of heterozygous factor V Leiden mutation. Testing of family members subsequently revealed the presence of this same mutation in his mother and all three siblings, although there was no family history of stroke, hypercoagulability, or atypical headaches. The patient was started on low-molecular-weight heparin and later transitioned to apixaban. Progression of his headache and visual abnormalities led to the discovery of increased intracranial pressure as demonstrated by papilledema and characteristic findings on computed tomography scan. He was treated with acetazolamide with improvement of his symptoms. CVT is uncommon and can be a diagnostic challenge due to its atypical presentation. Clinicians should consider this diagnosis in patients with a subacute onset of atypical headache, especially when accompanied by seizures, focal neurological deficits, or altered consciousness.

## Introduction

Cerebral venous sinus thrombosis (CVT) is a rare but potentially life-threatening condition that presents with non-specific symptoms [1,2]. Here, we describe the case of a young, male patient who presented with headache and other non-specific symptoms. While CVT is more common in women, it is also associated with local infection and hypercoagulable conditions, including protein C and S deficiency, factor V Leiden mutation, anti-thrombin III deficiency, thrombophilia, vasculitis, and malignancy [2]. The purpose of this case report is to describe the association of CVT with a factor V Leiden mutation and the subsequent clinical outcomes. Without timely diagnosis and proper management, CVT can lead to hydrocephalus, intracranial hypertension, transtentorial herniation, and death (in rare cases) if left untreated.

## Case presentation

**Figure 1:**
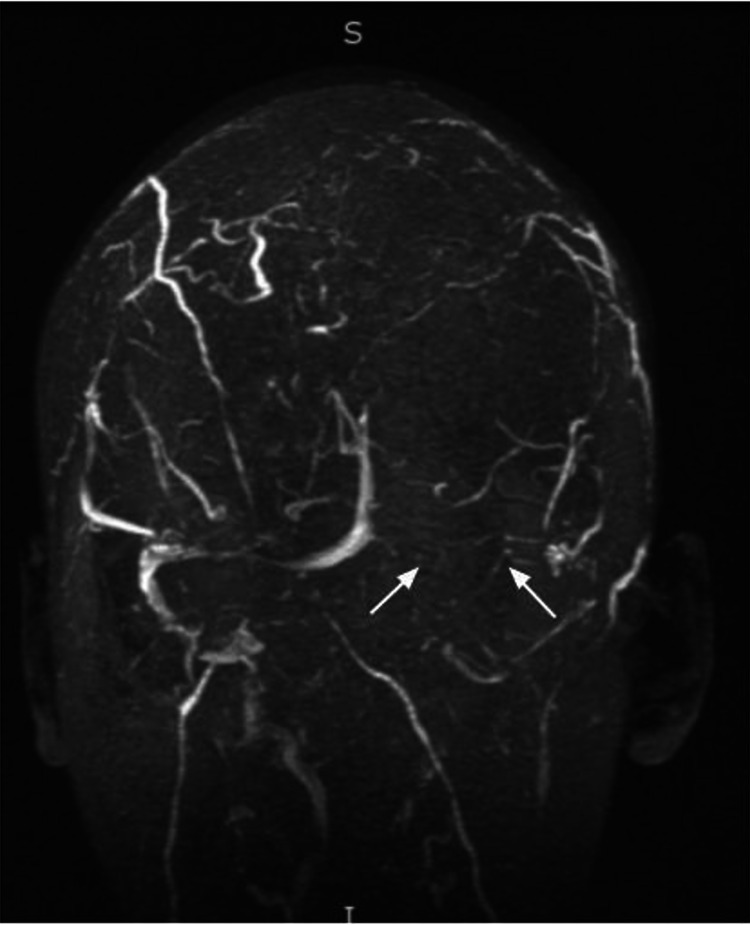
Left transverse sinus and left sigmoid sinus thrombus with arrows indicating lack of flow-related enhancement.

**Figure 2:**
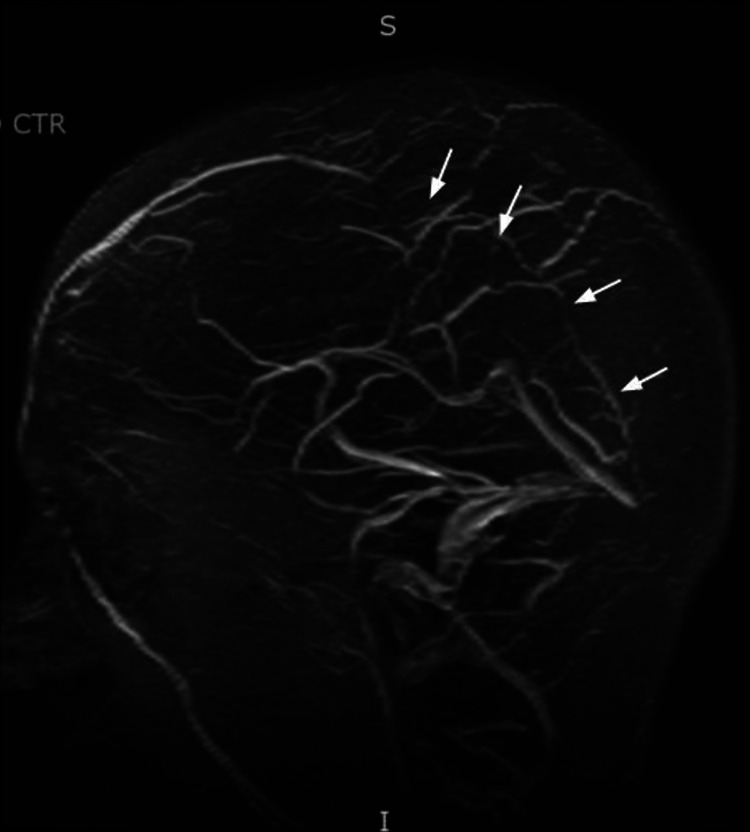
Arrows showing absent flow-related enhancement in the superior sagittal sinus consistent with thrombus.

A 24-year-old healthy male presented with a left temporal headache and non-specific, right-sided paresthesia. The headache developed in the left temporal area eight days prior. The patient initially attributed the symptoms to a migraine or temporomandibular joint syndrome, but the headache progressively worsened. Along with the headache, he experienced impaired vision (altered spatial sensation), bruxism, dental pain, nausea, and vomiting. After about a week, he developed paresthesia and apraxia in the right hand and paresthesia in the right lower extremity without loss of motor function and presented to the Emergency Department. The patient denied any other associated symptoms, including fever, chills, altered level of consciousness, seizure, or other alarming neurological symptoms. He denied any history of head injury, fall, stroke, drug use, or any known history of underlying hematological issues. He denied any similar symptoms previously. He had no known family history of stroke, venous thrombosis, bleeding disorders, or atypical migraine. The initial evaluation included a head computed tomography (CT) scan, which suggested superior sagittal venous thrombosis. This was confirmed with magnetic resonance imaging (MRI) and magnetic resonance venography (MRV), which also showed extensive thrombosis involving the superior sagittal, left transverse, left sigmoid, and left jugular venous sinuses, as well as superior cerebral cortical veins bilaterally (Figures 1, 2).

The patient was admitted for further evaluation and treatment. He was immediately started on anticoagulation with low-molecular-weight heparin and was later transitioned to apixaban. Extensive evaluation during his hospitalization revealed the presence of heterozygous factor V Leiden mutation R506Q. All other studies for hypercoagulability were negative. He was discharged from the hospital as his symptoms improved. At his follow-up visit two weeks later, he still had significant activity-limiting symptoms, including fatigue, headache, and persistent paresthesia. He was found to have papilledema on examination, and a repeat MRI confirmed evidence of increased intracranial pressure. This occurred despite improvement in thrombotic changes. His symptoms finally improved after starting acetazolamide, and a subsequent MRI showed gradual resolution of the thrombosis and recanalization of the venous sinuses. Subsequent evaluation of the patient’s family members revealed his mother and siblings, all three younger than him, to be positive for the same heterozygous factor V Leiden mutation. The patient’s father tested negative.

## Discussion

CVT is an uncommon disorder, with an annual incidence of about five in one million. It accounts for less than 1% of all strokes [1]. Most strokes due to CVT present with thromboses in multiple locations, particularly the transverse and sagittal sinuses, as in our patient. CVT is most common in those age 20-50, and it is three-fold more common in women than men, likely as a result of sex-specific risk factors such as contraceptive use and pregnancy. Other risk factors include protein C and S deficiency, factor V Leiden mutation, anti-thrombin III deficiency, thrombophilia, vasculitis, malignancy, inflammatory disorders, local infection, anemia, and head trauma [2]. CVT commonly presents as a non-specific headache in 60-90% of the patients [2]. The second most common presentation is new-onset seizures in 30-40% of the patients [3]. The headache associated with CVT may present in different ways, with no distinct pattern, and can mimic migraine, thunderclap headache, orthostatic headache, or cluster headache. The headache onset is typically sub-acute, with throbbing global (rather than unilateral) pain. Associated symptoms may include nausea, vomiting, and photophobia or phonophobia. The key feature in differentiating CVT headache from other types of headache is the positional worsening of the headache, particularly with recumbent position and worsening on performing the Valsalva maneuver [4].

There are four clinical syndromes described for CVT which are related to the location of the thrombus and affected brain parenchyma. These include an isolated intracranial hypertension syndrome presenting as headache, papilledema, decreased visual acuity, and tinnitus. Second, thrombosis of the superficial venous system and parenchyma lesions that presents with diffuse focal neurological deficits and seizures. Third, thrombosis of the deep venous system that can lead to mental status changes, diffuse encephalopathy, or coma. Finally, patients with cavernous sinus thrombosis may have orbital pain, chemosis, proptosis, and ophthalmoplegia. The risk of venous thrombosis in patients with heterozygous factor V Leiden mutation is approximately five times higher than normal [5].

The diagnosis of CVT is confirmed with central nervous system imaging, including CT venography, MRI, MRV, and catheter angiography. CVT is a medical emergency and the mainstay of treatment is anticoagulation with heparin or low-molecular-weight heparin initially, with bridging to warfarin or other oral anticoagulants. Patients with hemorrhagic CVT without other contraindication for anticoagulation should also be treated with heparin [6]. In patients with intracranial hypertension, acetazolamide or furosemide can be considered. Patients who remain symptomatic despite medical treatment should be considered for endovascular or surgical decompression [1]. If left untreated, CVT can lead to hydrocephalus, intracranial hypertension, and transtentorial herniation.

The clinical course of CVT is variable and unpredictable. About 25% of patients deteriorate acutely. Death occurs in 4-5% of patients and is generally due to transtentorial herniation. Risk factors for poor outcomes are male gender, increasing age, hemorrhagic infarct, intracranial hemorrhage, and coma [2].

## Conclusions

CVT is a rare condition with non-specific symptoms that may be missed due its complex and atypical presentation. It should be considered in the differential diagnosis of patients with subacute headache accompanied by seizures, focal neurological deficits, or altered levels of consciousness. Patients with this rare condition should be evaluated with hypercoagulable workup including recent vaccination. As demonstrated in the present case, CVT often requires a multidisciplinary approach to provide a successful treatment and to prevent morbidity and mortality.
